# Specificity of Molecular Fragments Binding to S100B versus S100A1 as Identified by NMR and Site Identification by Ligand Competitive Saturation (SILCS)

**DOI:** 10.3390/molecules26020381

**Published:** 2021-01-13

**Authors:** Brianna D. Young, Wenbo Yu, Darex J. Vera Rodríguez, Kristen M. Varney, Alexander D. MacKerell, David J. Weber

**Affiliations:** 1 Department of Biochemistry and Molecular Biology, University of Maryland School of Medicine, 108 N. Greene St., Baltimore, MD 21201, USA; brianna.young@som.umaryland.edu (B.D.Y.); darex@email.unc.edu (D.J.V.R.); kvarney@som.umaryland.edu (K.M.V.); 2 Center for Biomolecular Therapeutics (CBT), Baltimore, MD 21201, USA; wyu@rx.umaryland.edu (W.Y.); alex@outerbanks.umaryland.edu (A.D.M.J.); 3 Computer-Aided Drug Design Center, Department of Pharmaceutical Sciences, School of Pharmacy, University of Maryland, Baltimore, MD 21201, USA; 4 Institute for Bioscience and Biotechnology Research (IBBR), Rockville, MD 20850, USA

**Keywords:** S100B, S100A1, calcium, NMR, SILCS

## Abstract

S100B, a biomarker of malignant melanoma, interacts with the p53 protein and diminishes its tumor suppressor function, which makes this S100 family member a promising therapeutic target for treating malignant melanoma. However, it is a challenge to design inhibitors that are specific for S100B in melanoma versus other S100-family members that are important for normal cellular activities. For example, S100A1 is most similar in sequence and structure to S100B, and this S100 protein is important for normal skeletal and cardiac muscle function. Therefore, a combination of NMR and computer aided drug design (CADD) was used to initiate the design of specific S100B inhibitors. Fragment-based screening by NMR, also termed “SAR by NMR,” is a well-established method, and was used to examine spectral perturbations in 2D [^1^H, ^15^N]-HSQC spectra of Ca^2+^-bound S100B and Ca^2+^-bound S100A1, side-by-side, and under identical conditions for comparison. Of the 1000 compounds screened, two were found to be specific for binding Ca^2+^-bound S100A1 and four were found to be specific for Ca^2+^-bound S100B, respectively. The NMR spectral perturbations observed in these six data sets were then used to model how each of these small molecule fragments showed specificity for one S100 versus the other using a CADD approach termed Site Identification by Ligand Competitive Saturation (SILCS). In summary, the combination of NMR and computational approaches provided insight into how S100A1 versus S100B bind small molecules specifically, which will enable improved drug design efforts to inhibit elevated S100B in melanoma. Such a fragment-based approach can be used generally to initiate the design of specific inhibitors for other highly homologous drug targets.

## 1. Introduction

S100 proteins are dimeric EF-hand proteins that exhibit diverse protein-protein interactions upon Ca^2+^-binding [1]. S100B, one of over twenty S100 family-members, contributes to tumorigenesis and cancer progression [1]. In cancer cells, Ca^2+^-bound S100B binds the p53 tumor suppressor protein, inhibiting p53 phosphorylation, tetramerization and subsequent tumor suppressive function by stimulating cell proliferation and migration while downregulating apoptosis and differentiation [1,2]. Elevated S100B expression is a clinical biomarker of malignant melanoma (MM) indicating advanced disease stage, poor therapeutic response, and low patient survival [3,4]. Therefore, inhibiting the S100B-p53 interaction is of great interest in MM and other cancers. 

S100A1 is an S100 family member expressed in heart muscle, skeletal muscle and in the brain [5]. Some of its protein targets overlap with those of S100B. For example, both proteins bind to a 12-residue peptide (TRTK12) derived from the actin-capping protein CapZ, but S100A1 also binds to an exclusive set of biologically important protein targets [6,7]. For example, in cardiac and skeletal muscle, S100A1 interacts with the ryanodine receptor to promote sarcoplasmic reticulum (SR) calcium release and S100A1, but not S100B, regulates protein kinase A signaling in muscle and the nervous system, to name a few [5,8,9]. Likewise, S100A1 is up-regulated in a disease-specific manner, so developing S100A1-specific inhibitors may aid in treating diabetes, neurological diseases, heart failure, and other cancers [5,10,11,12,13,14]. While S100A1 and S100B have different biological functions, they share 60% sequence identity and are structurally homologous, potentially making inhibitor design specific for binding a single S100 protein challenging.

**Figure 1:**
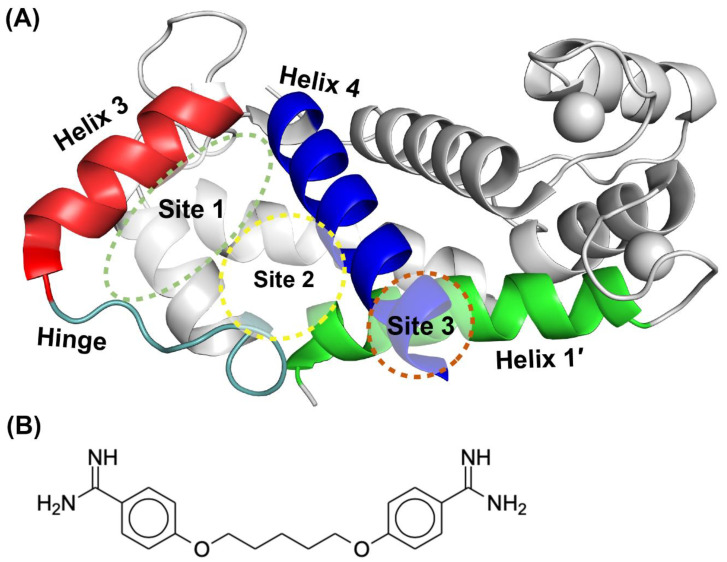
Three binding sites identified on S100B. (**A**) Site 1 (green dashed line) is comprised of Helix 3 (red), Helix 4 (blue) and the Hinge region (cyan). Inhibitors that lie in Site 2 (yellow dashed line) have interactions with residues within Helix 4 (blue) and the Hinge region (cyan). Site 3 (copper dashed line) interactions occur with residues of Helix 1′ (chain B, green), and Helix 4 (blue). (Figure adapted from literature) [16]. (**B**) Structure of the S100B inhibitor (SBi1), pentamidine.

As with other dimeric S100 proteins, each S100B subunit consists of a pseudo-EF hand and canonical EF hand that are linked by a loop termed the “hinge region” consisting of 10–12 residues [15]. Ca^2+^ binding by S100B leads to a conformational change in helix 3, resulting in exposure of a hydrophobic surface and facilitating target-binding [15]. S100B in the Ca^2+^-bound state has three binding pockets that have been exploited for drug-design purposes, termed Sites 1–3 (Figure 1A) [16]. Site 1 interactions were identified first using the structure of Ca^2+^-S100B complexed to the C-terminal regulatory domain of p53 [17]. This site is mostly comprised of residues from the hinge region and helices 3 and 4. Sites 2 and 3 were discovered originally via structural studies of S100B inhibitors (SBiXs) including the FDA approved drug pentamidine (SBi1) bound to Ca^2+^-S100B (Figure 1B) [18,19]. Interactions of Ca^2+^-S100B with pentamidine and other Site 2 inhibitors involve hydrophobic interactions with residues in loop 2 (F43, L44) and helix 4 (I80, A83, C84, F87, F88), which induced conformational changes to the Zn^2+^ binding site (H15, H25, H85, and E89) of S100B [19]. SBiXs that involve Site 3 typically involve hydrogen bonding between the small molecule and side chain moieties of D12 and E89 as well as with backbone atoms involving C84 and H85 [20]. For inhibitors interacting at this site, including SBi1, hydrophobic interactions with F88 and Ill are also known to be important [20].

A major goal in developing small molecule inhibitors of S100B is the identification of compounds that bind S100B with high affinity and a high degree of specificity, so multiple approaches were used. These include computer-aided drug design (CADD), NMR spectroscopy, X-ray crystallography, fluorescence spectroscopy-based high-throughput screening, and cell-based assays [21]. For example, pentamidine was discovered as an S100B inhibitor (SBi1) originally via computer aided drug design (CADD), in which hundreds of thousands of FDA approved drugs were screened computationally and then tested in vitro [22]. This drug has since been used for testing S100B inhibition in numerous disease states, including in a human clinical trial for malignant melanoma (0794GCC: “Treatment of Melanoma with Wild-type p53 and Detectable S100B Using Pentamidine: a Phase II Trial with Correlative Biomarker Endpoints”; NCT00729807) [23,24,25]. However, pentamidine is not an optimal cancer drug candidate since it is associated with toxicities on its own and when given in combination with numerous other medications. Pentamidine also binds to targets other than S100B including, S100 proteins such as S100A1, Calmodulin, PRL phosphatases, and DNA, which may cause off-target effects in vivo [5,26,27,28].

For these reasons, CADD and structure/activity relationship (SAR) studies were employed to engineer modifications of these SBiXs that improved S100B inhibitor binding affinity and specificity [29,30]. Despite the promising development of first generation S100B inhibitors, efficacy, specificity, and toxicity issues still warrant further drug screening, design, and investigation. 

Towards improving S100 protein specificity of the SBiXs, an NMR fragment-based screening approach was used to identify small molecule fragments that bind with either S100B or S100A1. NMR spectral perturbations of the two S100 proteins were analyzed upon small molecule binding including chemical shift perturbations (CSPs) and/or line-broadening effects (LBEs) for the fragments in the Maybridge library that did not cause aggregation (>99.9%). Subsequently, these NMR data together with a computational approach termed Site Identification by Ligand Competitive Saturation (SILCS), confirmed fragment specificity, and predicted binding modes for the compounds identified by NMR. While several compounds bound both S100 proteins in the presence of Ca^2+^, two bound Ca^2+^-S100A1 specifically and four bound to only Ca^2+^-S100B. These small molecule fragments now provide a basis for designing next stage S100 inhibitors, including for targeting S100B in melanoma in cancer. Likewise, the information derived here for Ca^2+^-S100A1 and Ca^2+^-S100B is relevant to targeting a variety of S100-protein associated diseases in a specific manner. 

## 2. Results

### 2.1. Fragment Compounds Specific for the Ca^2+^-Binding Proteins S100B or S100A1 were Identified Using NMR

**Figure 2:**
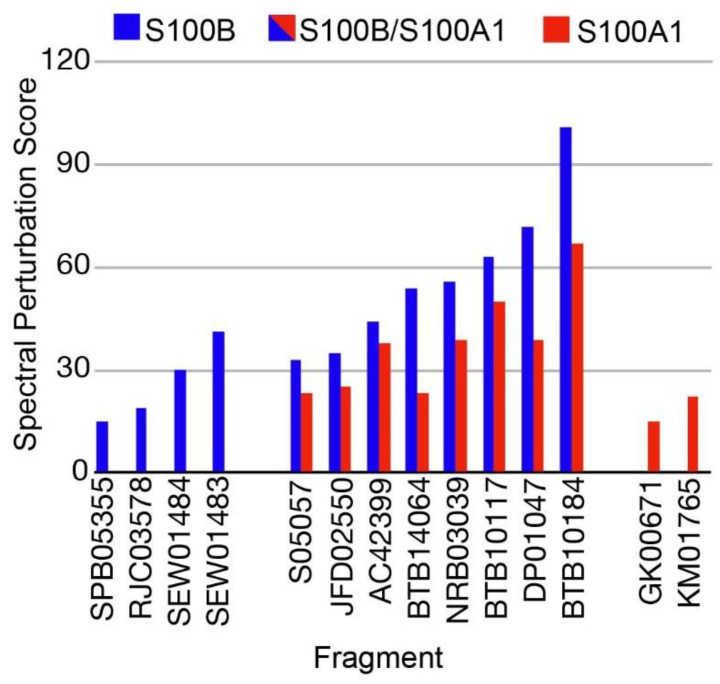
Maybridge Ro3 Fragments binding Ca^2+^-S100B and/or Ca^2+^-S100A1. Fragments that interact with either or both S100B (blue) and S100A1 (red). The method used to tabulate the spectral perturbations in the S100 proteins upon fragment addition is defined in Methods.

**Figure 3:**
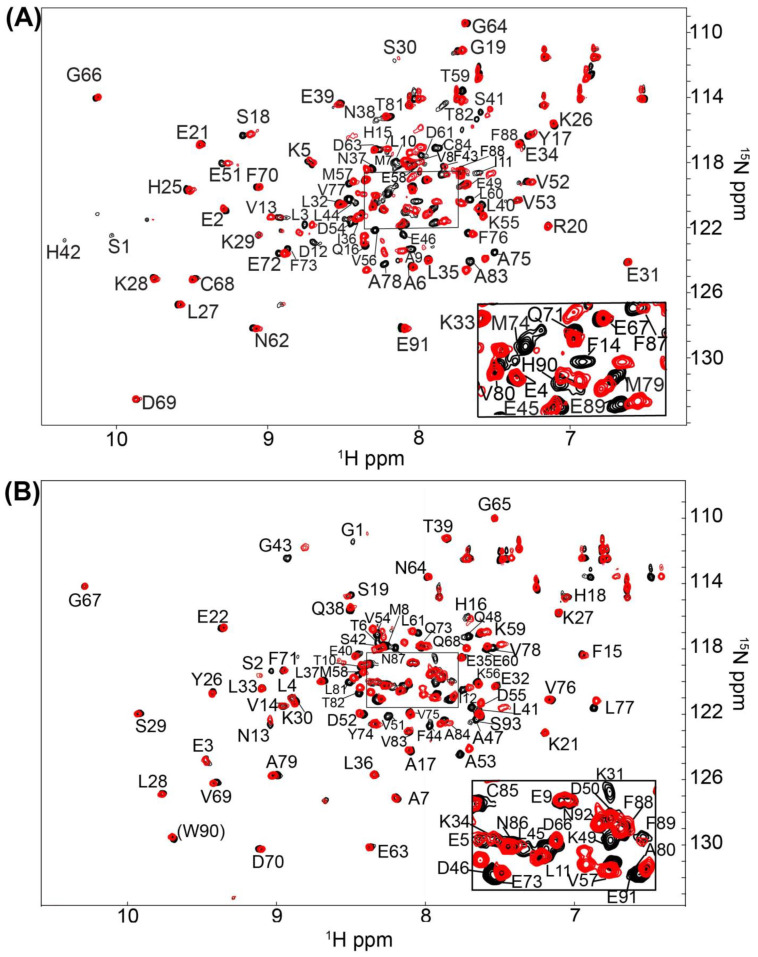
NMR data of S100B and S100A1 bound to the non-specific inhibitor BTB10184. 2D-[^1^H-^15^N] HSQC overlays of (**A**) Ca^2+^-S100B and (**B**) Ca^2+^-S100A1 with (red) and without (black) the nonspecific compound BTB10184. The NMR data was collected in the same manner for both S100 proteins under identical buffer conditions, at 800 MHz, 37 °C, as described in Methods.

**Figure 4:**
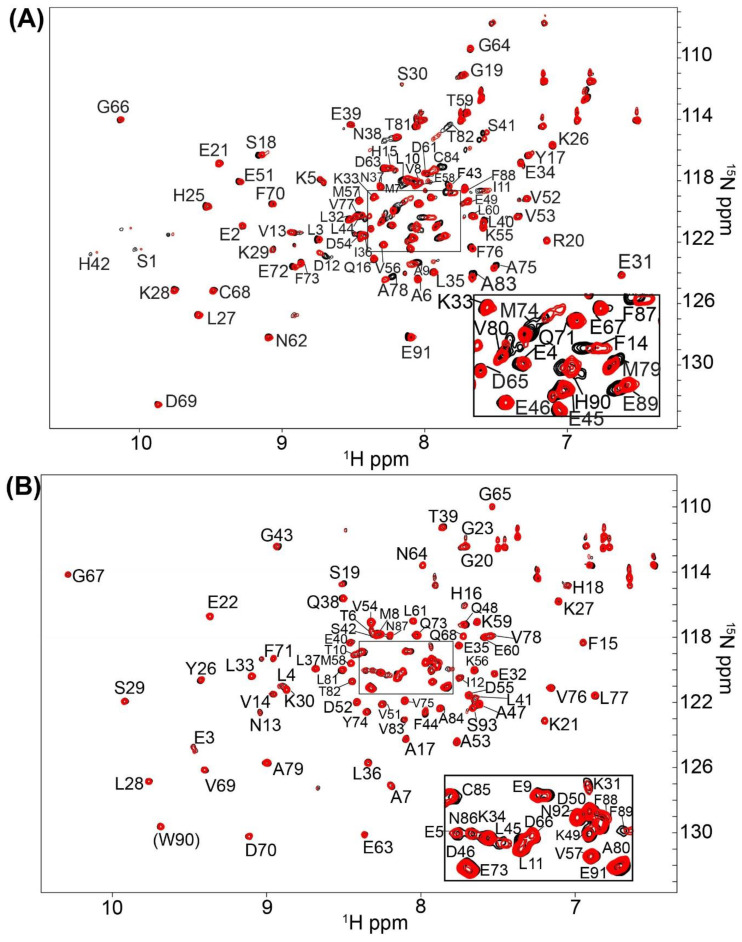
NMR data of S100B and S100A1 in the presence of the S100B specific inhibitor SEW01483. 2D-[^1^H, ^15^N]-HSQC overlays of (**A**) Ca^2+^-S100B and (**B**) Ca^2+^-S100A1 with (red) and without (black) the S100B-specific compound SEW01483. The NMR data was collected in the same manner for both S100 proteins under identical buffer conditions, at 800 MHz, 37 ^°^C, as described in Methods.

**Figure 5:**
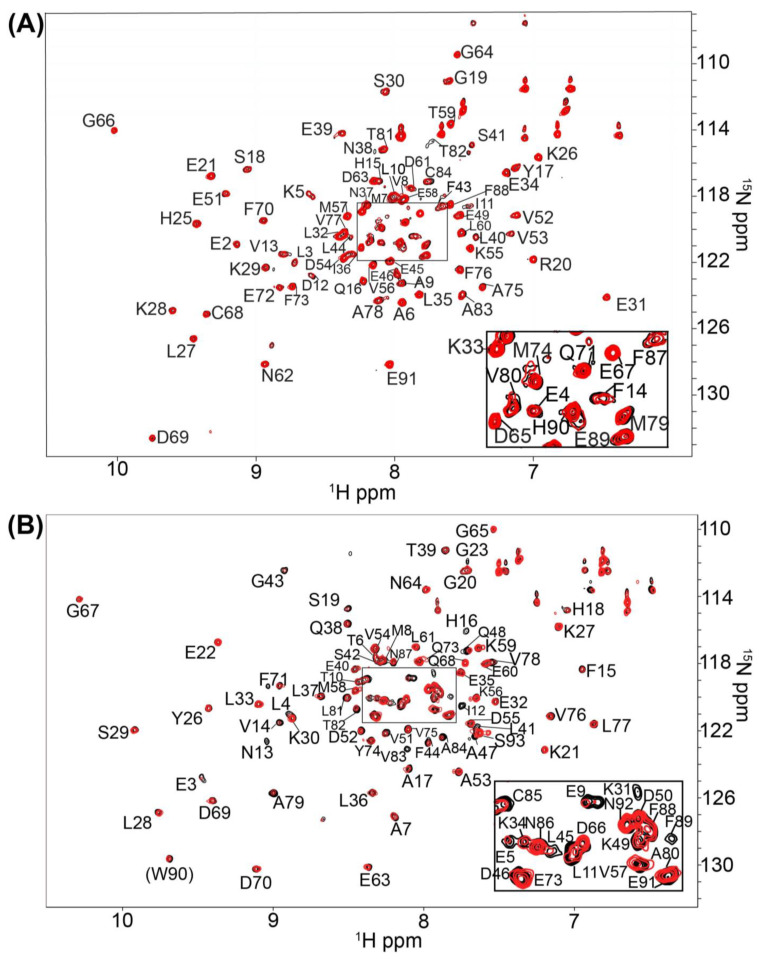
NMR data of S100B and S100A1 bound to the S100A1 specific inhibitor KM01765. 2D-[^1^H, ^15^N]-HSQC overlays of (**A**) Ca^2+^-S100B and (**B**) Ca^2+^-S100A1 with (red) and without (black) the S100A1-specific compound KM01765. The NMR data was collected in the same manner for both S100 proteins under identical buffer conditions, at 800 MHz, 37 °C, as described in Methods.

In an identical Ca^2+^-containing buffer, the 1000 compound Maybridge Ro3 fragment library was used to screen ^15^N-labeled S100B or ^15^N-labeled S100A1 by NMR with the goal of finding low molecular weight fragment compounds that are specific for each S100 protein. This compound library consists of 1000 structurally diverse highly soluble molecular fragments that have drug-like properties and reduced chemical complexity. The “rule of 3” used for fragment screening is typically followed in this library including the molecular fragments have (i) three or fewer hydrogen bond donors and acceptors; (ii) a ClogP value of 3 or less; (iii) a molecular weight of 300 Daltons or lower; (iv) fewer than 3 rotatable bonds, and (v) a polar surface area less 60 Å^2^ [31]. Spectral changes upon compound addition included chemical shift perturbations (CSPs) and/or line-broadening effects (LBEs) from fast- and/or intermediate exchange binding on the NMR chemical shift timescale. None of the small molecule fragments displayed binding in the slow-exchange regime [32]. Most of the compounds in the fragment library (>99%) did not perturb any ^H^N correlations in the 2D [^1^H, ^15^N]-HSQC NMR experiments for either Ca^2+^-bound S100A1 or Ca^2+^-bound S100B. Three fragments were found to cause extensive line-broadening in one or both S100 protein spectra due to aggregation, so data for these fragments were not used. Eight fragment compounds were nonspecific and found to perturb resonances within both ^15^N-labeled S100B and ^15^N-labeled S100A1, in the presence of Ca^2+^ (Figure 2). As a representative example, addition of BTB10184 induced LBEs and CSPs in spectra of both Ca^2+^-S100A1 and Ca^2+^-S100B (Figure 3). Importantly, two compounds provided spectral perturbations only to the NMR spectrum of Ca^2+^-bound S100A1, and four compounds provided perturbations only to Ca^2+^-bound S100B NMR data. Fragment SEW01483 is representative of an S100B-specific binding fragment since it showed notable changes in the [^1^H, ^15^N]-HSQC NMR spectrum of Ca^2+^-bound S100B but no NMR spectral changes to Ca^2+^-bound S100A1 (Figure 4A,B). Specifically, the ^H^N correlations of A9, I11, D12, F14, S41, F43, A78, A83 and C84 were perturbed upon addition of SEW01483 to Ca^2+^-bound S100B (Figure 4). On the other hand, the fragment compound KM01765 was found to perturb correlations in Ca^2+^-bound S100A1 with no observable effects to Ca^2+^-S100B. For this S100A1-specific fragment, the largest perturbations were observed for ^H^N correlations of I12 and residues in helix 4 and the C-terminal loop, including V78, V83, A84, and F89 (Figure 5). Several ^H^N correlations of Ca^2+^-S100A1 were found to disappear in the NMR spectrum upon KM01765 addition (I12, N13, H16, K31, V83, and F89), indicative of binding in the intermediate time regime on the NMR chemical shift scale (Figure 5). 

### 2.2. Site Identification by Ligand Competitve Saturation (SILCS) Studies of S100A1 and S100B in the Ca^2+^-Bound States

**Figure 6:**
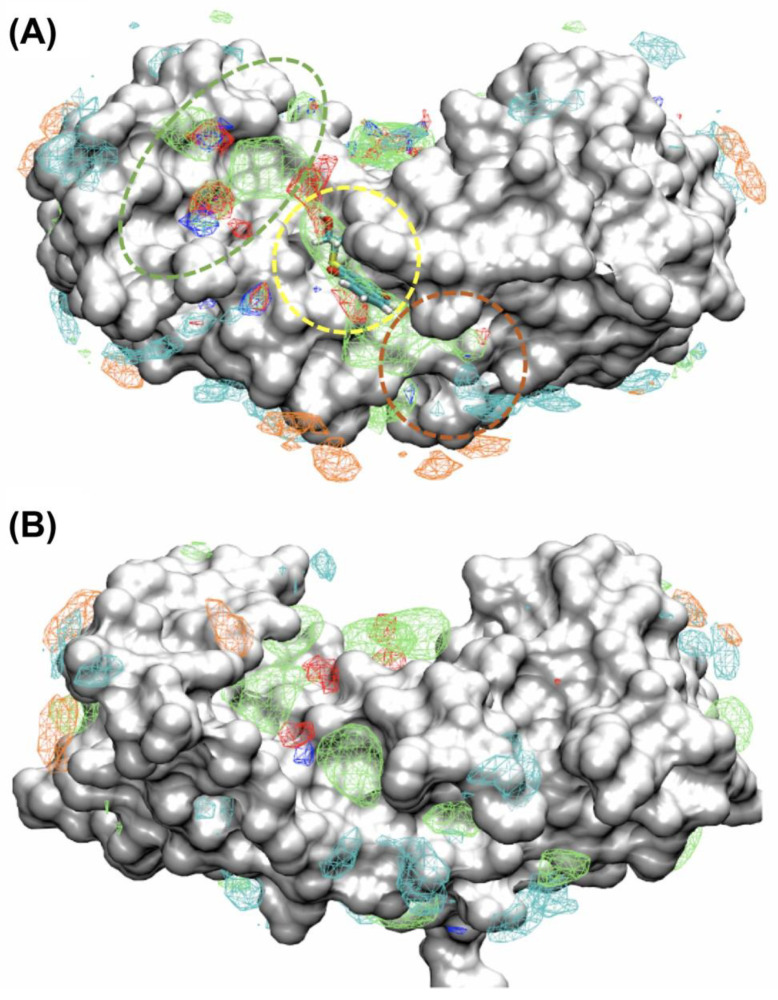
SILCS FragMaps of S100B and S100A1. SILCS FragMaps for (**A**) Ca^2+^-S100B and (**B**) Ca^2+^-S100A1. Maps are rendered at GFE level of −1.2 kcal/mol for Apolar (green), hydrogen bonding donor (blue) and acceptor (red) maps and at −1.5 kcal/mol for positively charged MAMN (cyan) and negatively charged ACEO (orange) maps. For S100B, Sites 1, 2 and 3 are shown in dashed circles in green, yellow, and copper, respectively [16]. The S100B inhibitor from the crystal structure 4PE0, which is located within the Site 2, is shown in (**A**) as a cyan stick representation.

To predict and map binding patterns of small molecules for Ca^2+^-bound S100A1 and S100B, SILCS FragMaps were calculated first, as shown (Figure 6). These FragMaps identify regions where apolar, hydrogen bond, and ionic interactions of small molecules with Ca^2+^-S100A1 and Ca^2+^-S100B may occur. For S100B, it was found that the hydrophobic binding mode of the inhibitor SBi4434 in the crystal structure 4PE0 was reproduced with the apolar FragMaps at Site 2 (Figure 6A). Consistent with previous studies, apolar FragMaps indicate a hydrophobic groove encompassing Sites 1, 2 and 3. The structure of Ca^2+^-bound S100A1 was then aligned with Ca^2+^-bound S100B to directly compare their binding patterns (Figure 6B). Comparable to their structural similarity, similar apolar binding patterns were seen along the groove on Ca^2+^-bound S100A1 as indicated by the apolar FragMaps, though differences in the shapes of the maps are evident. In the supplementary information Figure S1, difference maps between FragMaps of S100B and S100A1 are shown. It is obvious from these difference maps that at Site 1, S100B has more favorable binding patterns from apolar and hydrogen bonding acceptor and donor types than S100A1. Similarly, S100B also has favorable apolar binding ability compared to S100A1 at Site 3. However at Site 2, S100A1 has more favorable bindings from apolar and hydrogen bonding acceptor and donor types compared to S100B. Such information can be used to design specific binders and helps explain the specificity of the fragments to the two proteins, as identified by NMR.

### 2.3. Binding Sites on Ca^2+^-Bound S100A1 and S100B Predicted by SILCS-Hotspots

SILCS-Hotspots analyses were conducted to explore all potential fragment binding sites on Ca^2+^-bound S100B and S100A1. Figure S2 in the supplementary material shows all potential binding sites for the studied fragments on S100B and S100A1. Sites 1, 2, 3 and a site at the dimer interface were found to be “highly ranked” as fragment binding sites for both Ca^2+^-S100B and Ca^2+^-S100A1. This is consistent with the NMR chemical shift results, which show large spectral changes for numerous residues within Sites 1, 2, 3, as well as for residues in the dimer interface.

### 2.4. SILCS-MC Docking Poses and Lgfes Provide Insight into Fragment Specificity

**Figure 7:**
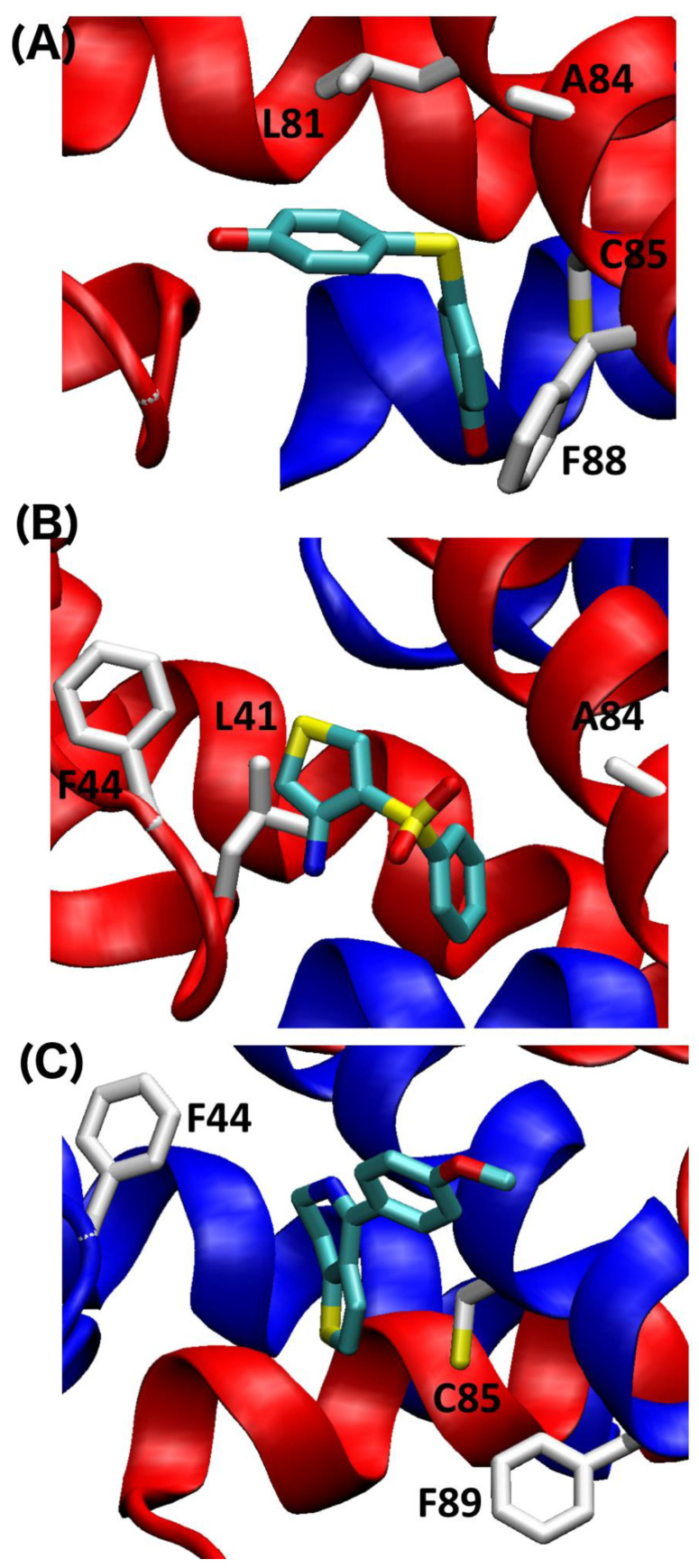
Predicted binding mode of (**A**) BTB10184 (**B**) KM01765 and (**C**) SEW01483 for Ca^2+^-S100A1. Residues in the pocket with large spectral perturbations are labeled. The two subunits of the S100 protein are colored in blue and red, respectively.

Three fragments were chosen for further analyses based on SILCS-Hotspots analysis and the NMR data. These include one fragment specific for S100A1 (KM01765), one specific for S100B (SEW01483), and one that binds both S100A1 and S100B (BTB10184) in the presence of Ca^2+^. The docking pose from SILCS-Hotspots that was most consistent with largest NMR spectral perturbations was used to represent the binding orientation for each fragment. For example, for SEW01483 bound to S100B, residues A9, I11, D12, F14, S41, F43, E45, A78, A83 and C84 show significant changes in the NMR data, and a docking pose from SILCS-Hotspots was found near four residues out of the ten (I11, D12, F43, C84), and thus selected for further analysis. LGFEs, which are an estimate of the binding free energy, were calculated for the selected binding pose for each fragment compound targeting S100A1 and S100B (Table 1). Consistent with the NMR findings, LGFE ranked the binding strength to Ca^2+^-bound S100A1 in the order of KM01765 > BTB10184 > SEW01483, though their binding differences are underestimated. Based on SILCS-MC and NMR perturbations, Site 2 was predicted to be the best binding site for all three fragments binding to S100A1, which is also consistent with the findings above based on SILCS difference maps. The best specific binder of S100A1 was found to be KM01765, which has interactions with Site 2 residues, including hydrophobic contacts with NMR correlations that are most perturbed including L41, F44, and A84 (Figure 7). The dual-binder BTB10184 was predicted to be the best binder to S100A1 and has formed the most hydrophobic contacts with Site 2 residues including L81, A84, C85 and F88, which have the largest NMR spectral perturbations (Figure 7). For SEW01483, the least contacts with Site 2 residues were observed, but affected residues are F44, C85 and F89, as indicated by the very small shifts in the NMR correlations (Figure 4 and Figure 7).

**Figure 8:**
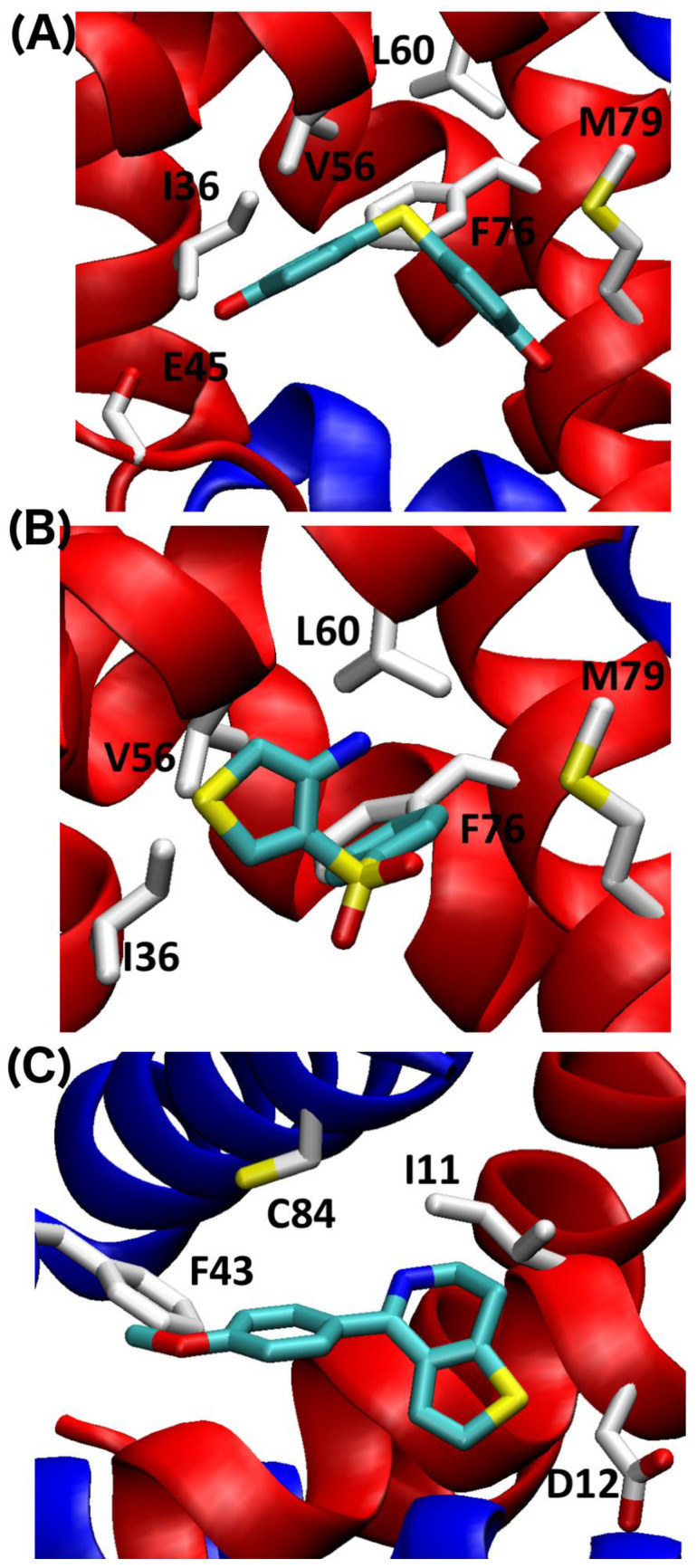
Predicted binding mode of (**A**) BTB10184 (**B**) KM01765 and (**C**) SEW01483 for Ca^2+^-S100B. Residues at the pocket are labeled. For (**C**), interface residues are colored by the chain color.

For S100B, the predicted binding strength of the three fragments is in the order of BTB10184 > SEW01483 > KM01765, which is also consistent with the NMR perturbation results showing that BTB10184 perturbs S100B the most while KM01765 shows no signs of spectral perturbation (Table 1). The much weaker predicted binding affinity for KM01765 is consistent with experimental findings indicating that it is specific to S100A1, not S100B. The binding poses for these fragments were predicted and illustrated in Figure 8. The best binding orientation of BTB10184 and KM01765 is at Site 1 of S100B, while the best orientation of SEW01483 is at Site 3 (Figure 8). The predicted binding mode of KM01765 to S100B suggests only a few hydrophobic contacts are formed between KM01765 and surrounding residues including V56 and M79, making it the weakest binder among the three (Figure 8). In contrast, BTB10184 forms well-defined hydrophobic contacts with residues I36, L44, V56, L60, and M79, which is consistent with strong NMR perturbations observed for these residues (Figure 3 and Figure 8). In addition, the two terminal hydroxyl groups can form hydrogen bonding with backbone carbonyl groups in residues E45 and M79, which is also confirmed by large NMR CSPs (Figure 3). All these interactions with Site 1 residues make fragment BTB10184 the best binder to S100B among the three. For SEW01483, the best-predicted binding pose is at Site 3 surrounded by residues Ill, D12, F43, and C84, which is consistent with the NMR results showing that these residues have large spectral changes (Figure 4 and Figure 8C). S100B forms more favorable hydrophobic contacts to SEW01483 compared to S100A1, which makes it a strong, specific binder to S100B (Figure 4). 

Consistent with NMR, LGFE predicts the fragment specificity over S100B and S100A1 satisfactorily (Table 1). KM01765 has a much more favorable LGFE value for S100A1 compared to S100B. In contrast, SEW01483 has lower LGFE values for S100B compared to S100A1. This is consistent with specificity found here by NMR. For the dual-binder BTB10184, the LGFE score shows that it favors binding to S100B over S100A1, and this is consistent with its much stronger perturbation results for S100B over S100A1. 

## 3. Discussion

While many compounds bind both S100A1 and S100B, finding a compound with specificity for either protein is challenging since these proteins share 60% sequence similarity and are structurally homologous (Figure 2). It is encouraging that small compounds were discovered that showed specificity for these two S100 proteins, since these novel findings may help overcome ongoing issues with toxicity associated with pentamidine and other S100B inhibitors under development. Specifically, fragment-based screening using the Maybridge Ro3 1000 fragment library was sufficient to identify two fragments that are S100A1-specific and four that are S100B-specific. Based on LGFEs and predicted binding poses, BTB10184 was predicted to bind both Ca^2+^-S100B and Ca^2+^-S100A1, which matches its lack of specificity and the significant spectral perturbations throughout both proteins upon binding, as observed by NMR. The computationally predicted binding strength of SEW01483 for S100B and S100A1 is also consistent with the NMR results that this compound is S100B specific. Similarly, the predicted binding strength of KM01765 agrees with the NMR results that this fragment has specificity for Ca^2+^-S100A1. 

Although BTB10184 bound to both S100A1 and S100B, it is predicted to interact in a different mode for the two proteins. Based on NMR and computational results, BTB10184 was the strongest binder to S100B of the three compounds examined. Consistent with NMR perturbation data, this non-specific compound is predicted to bind S100A1 at hydrophobic, Site 2 residues L81, A84, C85 and F88. However, computational and NMR results indicate it interacts with hydrophobic residues within Site 1 of S100B while also forming hydrogen bonds with backbone carbonyls of E45 and M79. As evidenced by large spectral perturbations, compounds that bind both proteins may do so at more than one site. Alternatively, these molecules may interact with many residues within a single site, causing some rearrangement of the overall protein structure. Fragments specific for either protein had lower overall CSP scores, suggesting that they may only bind a single site or interact with fewer residues overall. 

Based on NMR and SILCS-MC, SEW01483 is an S100B specific inhibitor that interacts with residues in Site 3 of Ca^2+^-S100B including with residues Ill, D12, F43, and C84. To further check its specificity, GFE contributions from atoms to the total LGFE for S100B and S100A1 were compared and are shown in Figure S3. Overall, GFE contributions from apolar atoms in SEW01483 are larger for Ca^2+^-S100B compared to Ca^2+^-S100A1. Therefore, the specificity is likely occurring from the more hydrophobic features in Site 3 on Ca^2+^-S100B compared to Site 2 on Ca^2+^-S100A1. SEW01483 and the other S100B-specific compounds discovered here provide a foundation for designing new S100B inhibitors. Linking the S100B-specific compound SEW01483 to another S100B specific fragment might provide both the specificity and efficacy needed to inhibit p53 binding to S100B. This compound might also be improved by linking it to the strongest binder of S100B observed by NMR, BTB10184. This might allow for inhibition of Site 1, the p53 binding-site, while also providing S100B specificity.

For S100A1 binding KM01765, interactions occur with Site 2 residues including hydrophobic contacts with residues L41, F44 and A84. This pocket might be a useful location for future drug design for Ca^2+^-S100A1. Shown in Figure S4 is a comparison between GFE contributions from atoms in KM01765 for Ca^2+^-S100B and Ca^2+^-S100A1. Contributions from apolar atoms to the total LGFE for S100B and S100A1 are similar. While GFE contributions from hydrogen bond donor and acceptors differ, the largest differences were for Ca^2+^-S100B versus Ca^2+^-S100A1 with a total of 0.4 kcal/mol difference in LGFE. This observation indicates compounds with more hydrogen bonding features might prefer S100A1 over S100B, which is also consistent with the following analysis based on chemical properties.

The chemical properties of the compounds specific for Ca^2+^-S100A1 and Ca^2+^-S100B were compared next. The S100B-specific compounds have a lower polar surface area (53 Å^2^), compared to S100A1 specific compounds that have an average polar surface area of 89 Å^2^ (Table 2). S100A1-specific compounds also have one more hydrogen bond acceptor compared to those specific for S100B. Consistent with this, SEW01484 binding to Ca^2+^-S100B results in a lower amount of spectral perturbations compared to that of SEW01483 and also results in minor perturbations of Ca^2+^-S100A1 (Figure 2). The only difference between the two fragments is that SEW01484 has an extra hydrogen bond donor, as it has a secondary amine, compared to SEW01483, which does not donate hydrogen bonds as a tertiary amine (Table 2). 

The methods employed here allowed for discovery of compounds specific for two closely related S100 proteins, Ca^2+^-S100B and Ca^2+^-S100A1. Additional insight was gained into why these compounds are specific to each protein based on their predicted binding pose. We conclude that hydrophobic heterocyclic fragments such as SEW01483 that lack strong hydrogen bonding donor groups such as amides, may show specificity for Ca^2+^-S100B over Ca^2+^-S100A1. While a fragment such as KM01765, which has hydrophobicity and additional strong hydrogen donor groups, may show specificity for Ca^2+^-S100A1 over Ca^2+^-S100B. A fragment like BTB10184, which has hydrophobic moieties and hydrogen donor groups may bind both Ca^2+^-S100A1 and Ca^2+^-S100B. Based on these results, work will continue towards resolving toxicity, efficacy and specificity issues observed with next generation S100B inhibitors. Improving upon the existing compounds will facilitate better design of drugs to target S100B in cancer. Furthermore, using the knowledge regarding specificity gained here will provide a foundation for further development of S100-protein specific inhibitors that can be used for a variety of diseases.

## 4. Materials and Methods 

### 4.1. Materials

D_6_-DMSO, D_2_O, ^15^NH_4_Cl, and ^13^C-glucose were purchased from Cambridge Isotopes Laboratories (Woburn, MA, USA). Other materials were of the highest commercial quality and passed through Chelex-100 resin (Bio-Rad, Hercules, CA, USA) to remove trace metals. The Maybridge Ro3 1000 Fragment Library was supplied through Thermo Fisher Scientific (Waltham, MA, USA). 

### 4.2. Sample Preparation

^15^N-labeled human S100B and ^15^N-labeled human S100A1 or ^15^N, ^13^C-labeled human S100B was over-expressed and purified from *Escherichia coli* as previously described for rat S100B [33]. Compounds from the Maybridge Ro3 1000 fragment library were solubilized to 200 mM in D_6_-DMSO with a Biomek FX liquid handler (Beckman Coulter, Brea, CA, USA). Compounds that remained insoluble via several techniques, including warming to 37 °C, sonication, and/or addition of D_6_-DMSO were diluted to 100 mM. NMR samples for the pooled and individual screens contained 100 µM human S100B or S100A1, 0.6 mM fragment (or a pool of 16 fragments), 10 mM HEPES pH 7.2, 15 mM NaCl, 0.30 mM NaN_3_, 0.20 mM TPEN, 5.0 mM DTT, 10 mM CaCl_2_, 0.2% EtOH, 5% D_6_-DMSO, 20% D_2_O. NMR assignments for human S100B were confirmed using standard multidimensional heteronuclear NMR techniques in this identical NMR buffer only with 1.0 mM [^13^C, ^15^N]-human S100B. 

### 4.3. Screening and NMR Data Collection

Fragment compounds from the Maybridge R03 library were screened first in pools of 16 compounds per sample by 2D [^1^H, ^15^N]-HSQC NMR experiments at 37 °C using an Avance 800 MHz (^1^H) NMR spectrometer equipped with a Bruker automatic sample changer (BACS 60) and automatic tune/lock capabilities (Bruker, Billerica, MA, USA). Samples of the pooled fragments that exhibited chemical shift perturbations in this preliminary screen were used to prepare samples containing only a single fragment per sample for final evaluation. The samples containing only 1 fragment per sample were examined using 2D [^1^H, ^15^N]-HSQC experiments at 37 °C using either the Bruker Avance 800 MHz (^1^H) and/or a Bruker Avance III 600 MHz NMR spectrometer, each equipped with four frequency channels and triple-resonance *z*-axis gradient 5 mm cryoprobes. 3D HNCACB data sets were collected to verify HSQC chemical shift assignments if/when needed. For rapid evaluation of fragment-dependent perturbations of 2D [^1^H, ^15^N]-HSQC spectra, spectral changes (SCs) were classified as either line-broadening effects (LBEs) or chemical shift perturbations (CSPs) and then subclassified and tabulated as weak (score = 1) or strong (score = 2) for each backbone ^1^H, ^15^N correlation based on the magnitude of the effect. LBEs were considered weak if the intensity of a 2D correlation was decreased by >40% (i.e., 40% < LBE < 90%) and strong if the correlation decreased by >90%. Fragments that induced considerable line-broadening throughout the entire spectrum (>30% of the ^H^N correlations), due to aggregation, were not considered further in such analyses and categorized separately as fragments that induced severe line broadening (Note: only 3 of 1000 were in this category; <99.9%). CSPs were tabulated as weak if their total perturbation $\surd \frac{1}{2}\left[\delta_{H}{}^{2}+{\left(0.14\times \delta_{N}\right)}^{2}\right]$ was (0.01 ppm < CSP < 0.025) and strong if their total chemical shift perturbation was ≥0.025 ppm upon summing the spectral change (SC) scores for all the perturbed correlations [34].

### 4.4. Structure Models for S100B and S100A1

Crystal structures were obtained through the protein data bank with PDB entry 4PE0 for S100B and 5K89 for S100A1 [35]. The structure of S100B chosen for this work (PDB: 4PE0) since the “FF-gate” in this structure of S100B is in the “open” position, as described previously, and the compound bound to Ca^2+^-S100B, SBi4434, was removed for all the computational studies [36]. For Ca^2+^-S100A1, homology modeling was conducted to provide a Ca^2+^-S100A1 model that has all 92 amino acid residues in both subunits for the simulations since residues between D47 and D53 on chain A and between A48 and V52 on chain C are missing in the X-ray crystal structure (5K89). SWISS-MODEL server was used to build homology models and the model with the best score was selected. The ligands both crystal structures were removed and Ca^2+^ ions were retained for all subsequent SILCS simulations with these two S100 proteins.

### 4.5. SILCS Simulation

Site Identification by Ligand Competitive Saturation (SILCS) simulation is used to explore functional group affinity patterns on both Ca^2+^-S100B and Ca^2+^-S100A1. The SILCS method involves molecular simulations of the target protein immersed in an aqueous solution that contains additional organic solutes of different chemical classes [37]. The solutes and water then compete for binding sites on the protein surface during the simulation, yielding a free energy fragment competition assay from which 3D fragment probability distributions of the solutes are used to define affinity patterns, termed FragMaps, encompassing a dynamic protein surface.

The current SILCS run was performed using the oscillating chemical potential Grand Canonical Monte Carlo (GCMC)/MD protocol for SILCS [38,39]. The structure models of S100B and S100A1 developed as described above was used to initialize the SILCS simulations. The protein was solvated in a water box, the size of which was determined to have the protein extrema separated from the box edge by 12 Å on all sides. Eight representative solutes with different chemical functionalities (benzene, propane, acetaldehyde, methanol, formamide, imidazole, acetate, and methylammonium) were added into the system at ~0.25 M concentration, to probe the functional group requirements of the protein. Ten such systems with different fragment positions were created to expedite the convergence of the simulations. Each system was minimized for 5000 steps with the steepest descent (SD) algorithm in the presence of periodic boundary conditions (PBC) and was followed by a 250 ps MD equilibration [40]. During SILCS simulations, weak restraints were applied on the backbone alpha carbon atoms with a force constant (k in 1/2 kδx^2^) of 0.12 kcal/mol/Å^2^ to limit large conformational changes in the protein and to prevent the rotation of the protein in the simulation box. The ten GCMC/MD simulations were run for 100 cycles where each cycle has 200,000 steps of GCMC and 1.0 ns of MD, yielding a cumulative 200 million steps of GCMC and 500 ns of MD. During GCMC, solutes and water are exchanged between their gas-phase reservoirs; the excess chemical potential used to drive such exchange is varied every 3 cycles to yield an average concentration corresponding to 0.25 M of each fragment [38]. The configuration at the end of each GCMC run is used as the starting configuration for the following MD. During MD, the Nosé−Hoover method was used to maintain the temperature at 298 K and pressure was maintained at 1 bar using the Parrinello−Rahman barostat [41,42,43]. CHARMM36m protein force field, CHARMM General Force Field (CGenFF) and modified TIP3P water model [44,45,46,47] were used to describe protein, organic solutes, and water during the simulation, respectively. GCMC was performed and MD was conducted using GROMACS program [39,48].

3D probability distributions of the selected atoms from the organic solutes, called “FragMaps,” from the SILCS simulations were constructed and combined to obtain both specific and generic FragMap types as previously described [49]. Atoms from snapshots output every 10 ps from each SILCS simulation trajectory were binned into 1 Å × 1 Å × 1 Å cubic volume elements (voxels) of a grid spanning the entire system to acquire the voxel occupancy for each FragMap atom type being counted. The voxel occupancies computed in the presence of the protein were divided by the value in bulk to obtain a normalized probability. Normalized distributions were then converted to grid free energy (GFE) based on a Boltzmann transformation for quantitative use [49].

### 4.6. SILCS-Hotspots Analyses

SILCS-Hotspots analyses were performed using SILCS FragMaps to identify potential binding sites globally on Ca^2+^-S100B and Ca^2+^-S100A1 for the NMR tested fragments [50]. The SILCS-Hotspots algorithm is based on fragment Monte Carlo (MC) sampling using SILCS FragMaps (SILCS-MC) [49]. The protein system was partitioned into a collection of 14.14 Å^3^ sampling boxes that covers the entire protein. In each sampling box, SILCS-MC was conducted from initially randomized fragment positions that were then subjected to 10,000 MC steps at 300K of molecular translations and rotations up to 1.0 Å and 180.0°, respectively, and rotation of dihedrals about rotatable bonds of up to 180.0°. This was followed by 40,000 MC simulated annealing steps from 300 to 0 K of molecular translations and rotations up to 0.2 Å and 9.0°, respectively, and rotation of dihedrals about rotatable bonds of up to 9.0°. This process was repeated 1000 times for each fragment in each sphere following which clustering was performed to remove redundancy and keep the representative binding pose per site. A second round of clustering was conducted next to identify Hotspots populated by one or more different fragments. 

## 5. Conclusions

**Figure 9:**
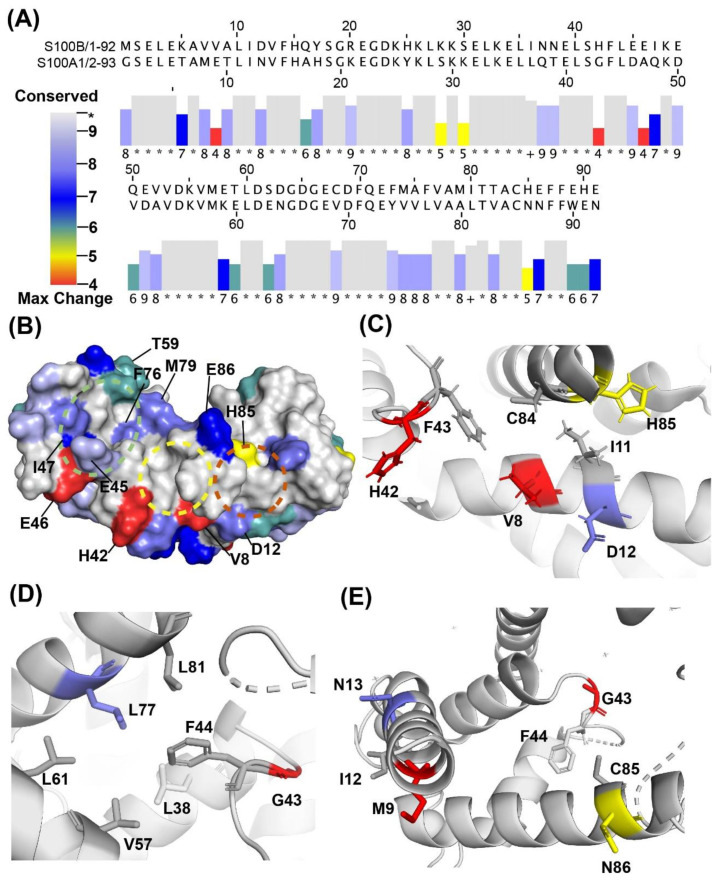
Sequence similarities and differences for S100B and S100A1. (**A**) Sequence alignment and conservation score of S100A1 and S100B calculated in JalView [51,52]. Columns with conserved residues are indicated by ‘*’, columns with residue changes where all properties are conserved are indicated by ‘+’, while columns with residue changes are marked with values ranging from ‘9’ to ‘4’ depending on the physiochemical properties of the changed amino acid residue. (**B**) Colors corresponding to the conservation scores were mapped onto a surface diagram of Ca^2+^-S100B (4PE0). Residues specific for S100B (scored 4–9) within Sites 1–3 are labeled. Sites 1, 2 and 3 are shown in dashed circles in green, yellow and copper, respectively. (**C**) Ribbon diagram of Site 3 of Ca^2+^-S100B (4PE0) with Site 3 residues shown in sticks. (**D**) Ribbon diagram of G43 and F44 in Ca^2+^-S100A1 (5K89) and (**E**) Ca^2+^-S100A1 amino acid residues corresponding to Site 3 of S100B shown in sticks. Colors correspond to the residue conservation score in (**A**).

It is important that S100B inhibitors designed to control malignant melanoma do not block S100A1, ensuring that normal skeletal and cardiac muscle function is retained. For designing inhibitors such as these, fragment-based approaches were applied including a combination of NMR and computer aided drug design. Here, the results with both S100A1- and S100B-specific fragments were discovered and provide insight into how to initiate the design of S100-protein specific inhibitors. For example, the S100B-specific fragment SEW01483, is shown to interact with Site 3 residues of S100B. Amino acid residues unique to S100B allow this pocket to form in S100B (Figure 9), but those interactions do not occur in S100A1. These methods can also be used generally to initiate the design of specific inhibitors for other highly homologous drug targets.

## Figures and Tables

**Table 1:** LGFEs for predicted binding poses of compounds for S100A1 and S100B.

Name	Specificity	S100A1 LGFE	S100B LGFE
BTB10184	Both	−6.52	−7.58
KM01765	S100A1	−6.14	−5.43
SEW01483	S100B	−5.97	−6.13

**Table 2:** Chemical properties of fragments that bind to either S100B or S100A1 specifically and a representative fragment (BTB10184) that binds to both proteins.

Compound	2D Structure	Specificity	H-Bond Donor	H-Bond Acceptor	Polar Surface Area (Å^2^) ^A^
SPB05355	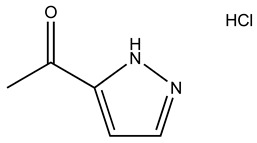	S100B	2	2	45.8
RJC03578	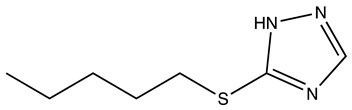	S100B	1	3	66.9
SEW01483	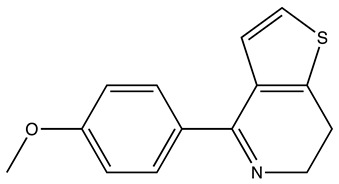	S100B	0	3	49.8
SEW01484	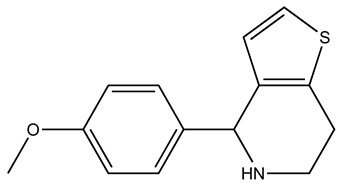	S100B	1	3	49.5
GK00671	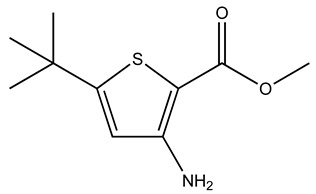	S100A1	1	4	80.6
KM01765	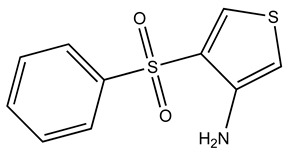	S100A1	1	4	96.8
BTB10184	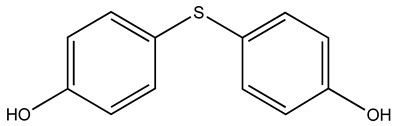	S100B/S100A1	2	3	65.8

^A^ Polar Surface Area (Å^2^) was computed by Cactvs 3.4.6.11 (PubChem release 18 June 2019).

## Data Availability

Data presented in this work are available on request from the corresponding author.

## Supplementary Materials

The following are available online, Figure S1: Difference maps for S100B and S100A1, Figure S2: SILCS-Hotspots analyses identified sites on (A) S100B and (B) S100A1, Figure S3: GFE contributions from atoms in SEW01483 to LGFE for (A) S100B and (B) S100A1, Figure S4. GFE contributions from atoms in KM01765 to LGFE for (A) S100B and (B) S100A1.

## Sample Availability

Samples of the compounds are not available from the authors. Compounds are available from Maybridge supplied by Thermo Fisher Scientific.
