# Supplementary material for: Specificity of Molecular Fragments Binding to S100B versus S100A1 as Identified by NMR and Site Identification by Ligand Competitive Saturation (SILCS)
Source: Molecules. 2021 Jan 13;26(2):381. doi: 10.3390/molecules26020381 (PMC7828390; doi:10.3390/molecules26020381)
Supplement: Supplementary file 1 [file molecules-26-00381-s001.pdf]

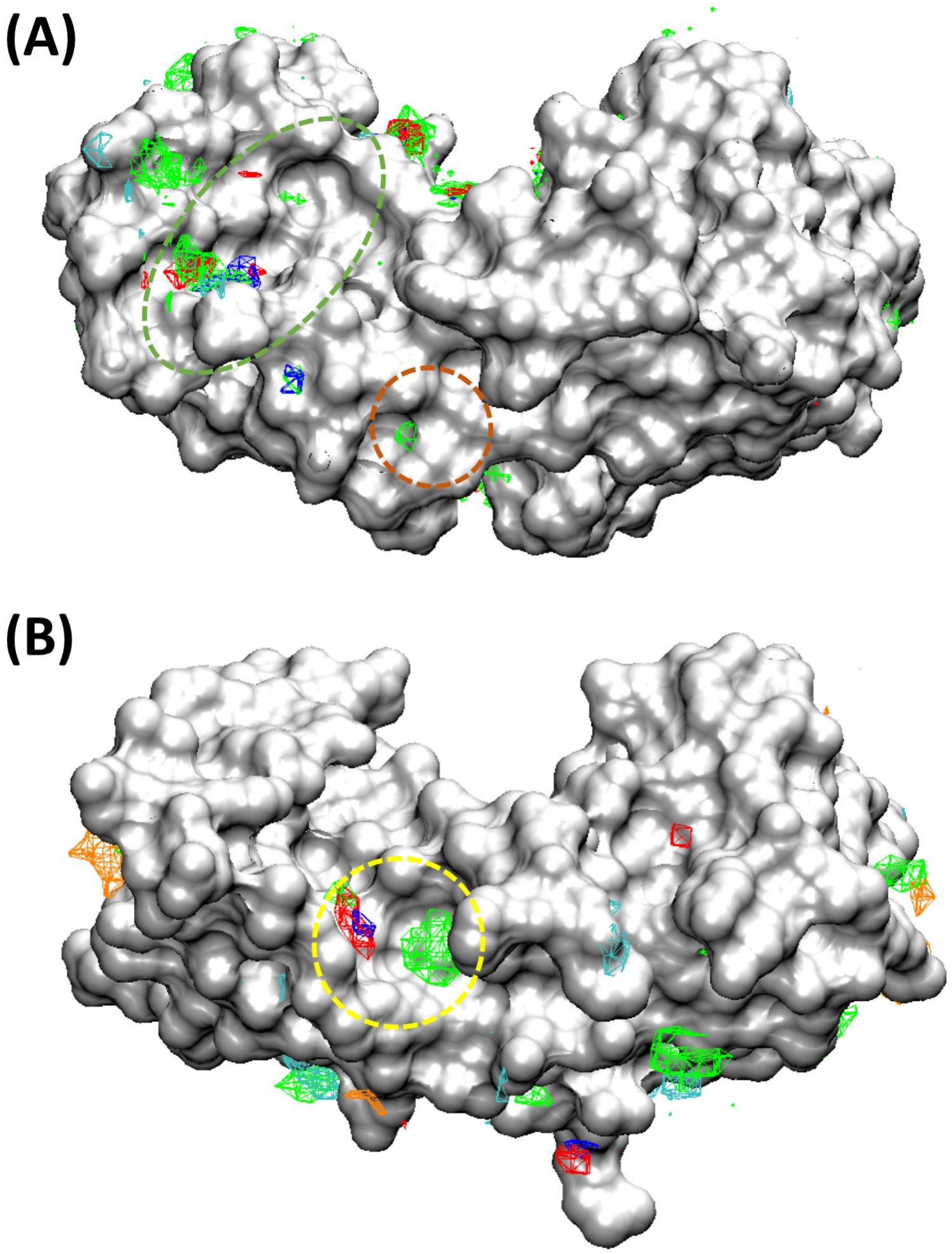

Figure S1. Difference SILCS FragMaps for S100B and S100A1. Binding patterns that are more favorable for S100B are shown in (A) by apolar (green), hydrogen bond donor (blue), acceptor (red), positively charged MAMN (cyan) and negatively charged ACEO (orange) difference maps. While those which are more favorable for S100A are shown in (B).

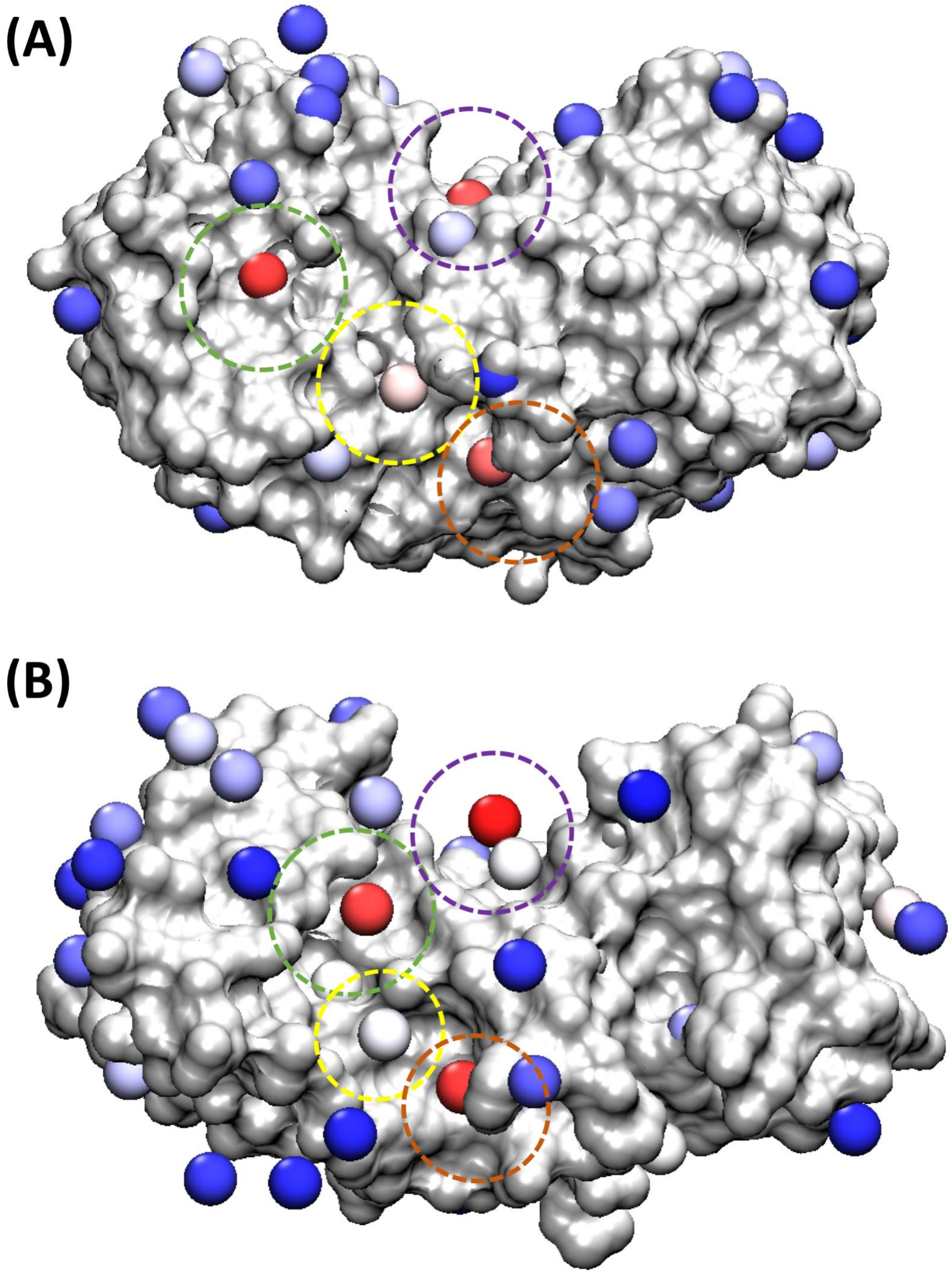

Figure S2. Predicted fragment binding sites from SILCS-Hotspots analyses on (A) S100B and (B) S100A1. Sites are ranked based on the average LGFE over all fragments in each site in a Red-White-Blue color scale with red being the most favorable. Top scoring sites are circled in green, yellow, red, and purple for Site 1, 2, 3 and the dimer interface.

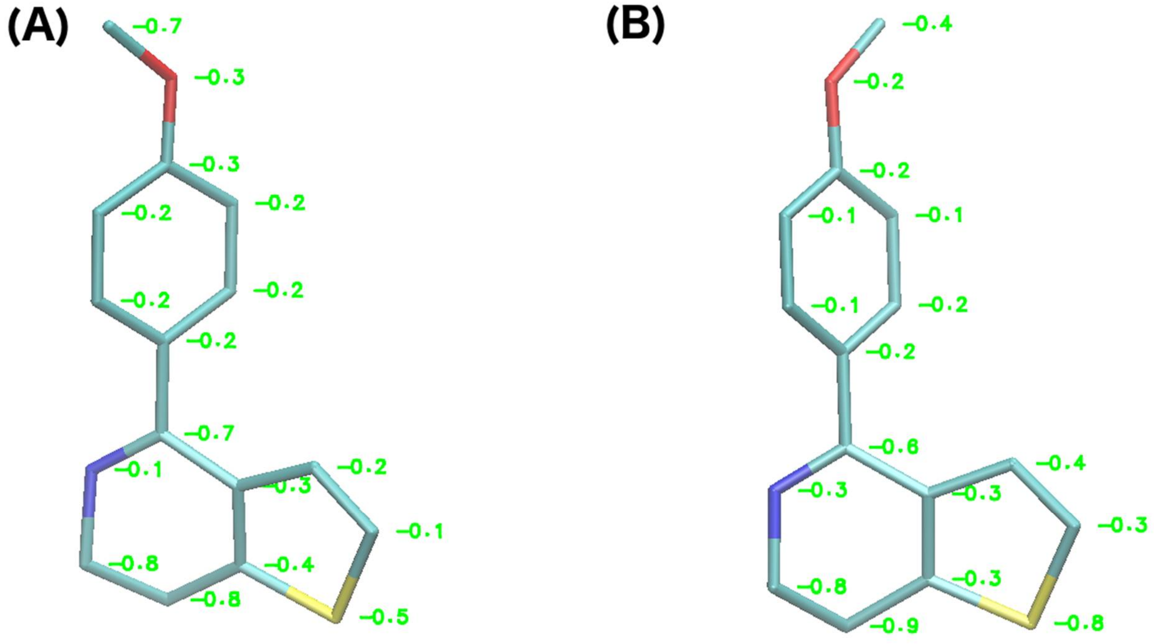

Figure S3. GFE contributions from atoms in SEW01483 to LGFE for (A) S100B and (B) S100A1.

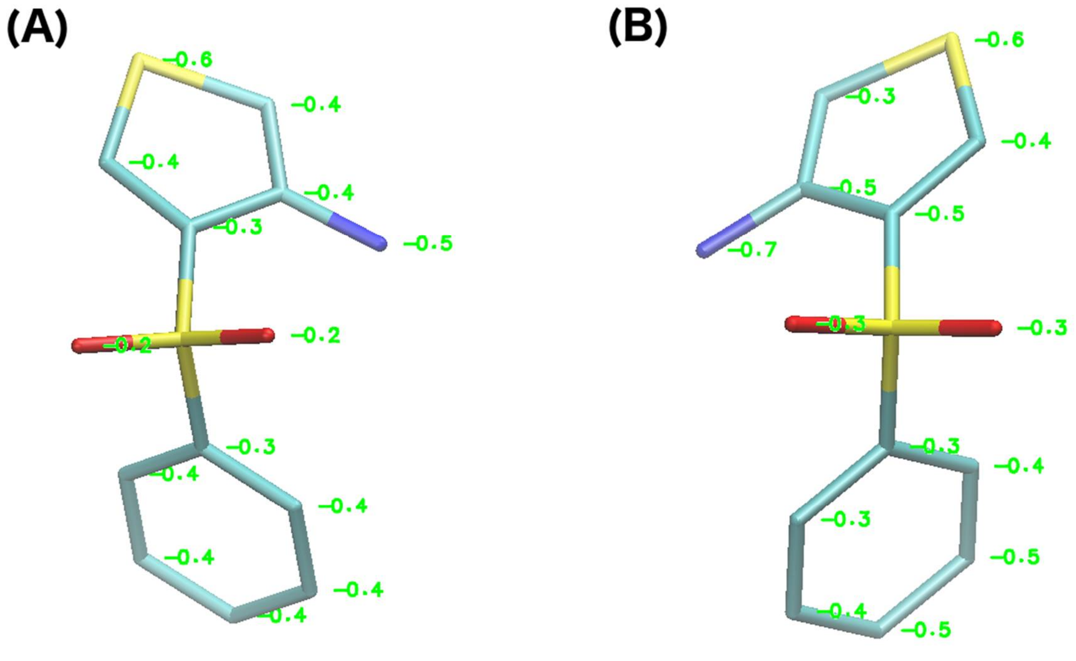

Figure S4. GFE contributions from atoms in KM01765 to LGFE for (A) S100B and (B) S100A1.
